# Optimism moderates psychophysiological responses to stress in older people with Type 2 diabetes

**DOI:** 10.1111/psyp.12806

**Published:** 2016-12-21

**Authors:** S. Puig‐Perez, R. A. Hackett, A. Salvador, A. Steptoe

**Affiliations:** ^1^ Department of Psychobiology and IDOCAL, Laboratory of Social Cognitive NeuroscienceUniversity of ValenciaValenciaSpain; ^2^ Department of Epidemiology and Public HealthUniversity College LondonLondonUK

**Keywords:** Type 2 diabetes, Optimism, Blood pressure, Cortisol, Psychological stress

## Abstract

Optimism is thought to be beneficial for health, and these effects may be mediated through modifications in psychophysiological stress reactivity. Type 2 diabetes (T2D) is associated with reduced cardiovascular responses to stress and heightened cortisol over the day. This study assessed the relationships between optimism, stress responsivity, and daily cortisol output in people with T2D. A total of 140 participants with T2D were exposed to laboratory stress. Heart rate (HR), systolic (SBP), diastolic blood pressure (DBP), and cortisol were measured throughout the session. Cortisol output over the day was also assessed. Optimism and self‐reported health were measured using the revised Life Orientation Test and the Short Form Health Survey. Optimism was associated with heightened SBP and DBP stress reactivity (*p*s < .047) and lower daily cortisol output (*p* = .04). Optimism was not related to HR, cortisol stress responses, or the cortisol awakening response (*p*s > .180). Low optimism was related to poorer self‐reported physical and mental health (*p*s < .01). Optimism could have a protective role in modulating stress‐related autonomic and neuroendocrine dysregulation in people with T2D.

There is increasing interest in psychological characteristics that could have a protective role in preventing the development of disease (Boehm & Kubzansky, 2012; Rasmussen, Scheier, & Greenhouse, 2009). Optimism is a psychological trait characterized by positive expectations about future outcomes that has been associated with better psychological and physical well‐being, particularly during times of stress (Scheier & Carver, 1992; Smith & MacKenzie, 2006). Optimism is thought to play a protective role in stress‐related conditions such as the metabolic syndrome (Cohen, Panguluri, Na, & Whooley, 2010), reduced immune functioning ( 2009; Roy et al., 2010), and cardiovascular diseases (Giltay, Kamphuts, Kabmijn, Zitman, & Kromhout, 2006; Nabi et al., 2010; Tindle, Davis, & Kuller, 2010).

The pathways underlying the potential protective effect of optimism on health remain unclear. Optimism may affect health indirectly through health behaviors, as well as directly through psychophysiological processes. Both of these pathways can influence the development of cardiovascular and other chronic diseases (Matthews, Räikkönen, Sutton‐Tyrrell, & Kuller, 2004; Tinker et al., 2007). There is considerable evidence that the protective role of optimism may involve in part greater engagement in health‐protective behaviors (Carver, Scheier, & Segerstrom, 2010). In line with behavioral self‐regulation theory (Carver & Scheier, 2000), the way in which people face challenges or difficulties influences how they cope with stress (Carver et al., 2010). Having an optimistic point of view increases confidence, motivating individuals to achieve goals as well as increasing positive affect and well‐being (Solberg Nes, Segerstrom, & Sephton, 2005). Optimism is linked with adaptive coping styles and health protective behaviors (e.g., such as better treatment adherence, lower consumption of saturated fat, increased vitamin intake, as well as increased physical activity; Giltay et al., 2006; Leedham, Meyerowitz, Muirhead, & Frist, 1995; Nabi et al., 2010; Shepperd, Maroto, & Pbert, 1996). However, associations between optimism and health outcomes in many studies persist after controlling for these factors, suggesting that other pathways may be involved.

The influence of optimism on psychophysiological processes involved in disease can be investigated using acute mental stress testing. Acute stress studies assess dynamic psychophysiological responses to stress under controlled conditions, which reduce the impact of possible confounding factors (Steptoe & Poole, 2010). Previous studies of healthy individuals have found that greater optimism is associated with increased heart rate (HR) stress responses (Solberg Nes et al., 2005), as well as faster cortisol recovery after stress (Brydon et al., 2009). However, in older people, associations have not been consistently detected. Endrighi, Hamer, and Steptoe (2011) failed to detect a relationship between optimism and cortisol responses to stress, while Puig‐Perez et al. (2015) found that older men and women with higher optimism had lower HR responses to acute psychosocial stress as well as a faster return to baseline levels posttask in cortisol and HR. It is possible that optimistic people demonstrate greater task engagement and this induces greater psychophysiological activation, resulting in heightened cardiovascular and endocrine responses (Solberg Nes et al., 2005). But at the same time, an optimistic perspective could protect the individual from exaggerated stress reactions, and thereby have a health‐protective effect (Puig‐Perez et al., 2015).

Given the relationship between acute stress responses and health, the study of factors that could have a protective role is important, especially in a vulnerable population such as people diagnosed with Type 2 diabetes (T2D). T2D is a heterogeneous metabolic disease characterized by reduced insulin sensitivity and relative insulin deficiency (International Diabetes Federation, 2015; McCrimmon, Ryan, & Frier, 2012). It is one of the most common diseases in people aged 50 and over (Scully, 2012) and is expected to continue to increase in prevalence in the coming decades (International Diabetes Federation, 2015; Zimmet, Alberti, & Shaw, 2001).

There is evidence that stress may play a role in the development of T2D (Hackett & Steptoe, 2016; Kelly & Ismail, 2015; Pouwer, Kupper, & Adriaanse, 2010). Meta‐analyses of prospective cohort studies suggest that people exposed to job strain (Nyberg et al., 2014) or who work long hours (Kivimäki et al., 2015) have a greater risk of developing T2D. Other longitudinal studies have also observed an increased risk of T2D in people with a history of moderate and severe childhood abuse (Rich‐Edwards et al., 2010) or childhood neglect (Goodwin & Stein, 2004). It has also been shown that higher perceived stress in adulthood increases the risk of T2D in a prospective 35‐year follow‐up study (Novak et al., 2013), although not all findings relating perceived stress with subsequent diabetes development have been consistent (Rod, Grønbaek, Schnohr, Prescott, & Kristensen, 2009; Williams, Magliano, Tapp, Oldenburg, & Shaw, 2013).

Stress‐related physiology has not been studied extensively in T2D, but one useful concept in this regard is allostasis, an adaptive process by which the body responds to changes in the environment through the adjustment of multiple biological systems (McEwen, 1998). Sustained or repeated exposure to challenge can result in chronic allostatic load and a breakdown of regulatory processes. McEwen (1998, 2006) has argued that high allostatic load is associated with blunted acute responses coupled with impaired poststress recovery. A trial carried out by our group suggests that physiological responses to stress may be dysregulated in people with T2D when compared with the responses of healthy controls, showing a pattern of responses characteristic of high allostatic load (Steptoe et al., 2014). Specifically, we observed that the participants with T2D had blunted stress reactivity and impaired recovery in blood pressure (BP), HR, cortisol, interleukin 6, and serum cholesterol in response to a laboratory stress task when compared with healthy controls (Steptoe et al., 2014).

It is worth noting that having a chronic disease such as T2D has been associated with a lower health‐related quality of life (Rothrock et al., 2010), and psychological distress is common in people with T2D (Hackett & Steptoe, 2016). Reduced quality of life is seen in a range of other conditions such as sarcoidosis (Wilsher, 2012) and breast cancer (Petersen et al., 2008) and is common following trauma (Tøien, Bredal, Skogstad, Myhren, & Ekeberg, 2011). According to Broffenbrenner's ecological model (McLeroy, Bibeau, Steckler, & Glanz, 1988), personality is a key influencer of quality of life. Accordingly, optimism is relevant as it may contribute to a better acceptance of living with a chronic disease such as T2D, thus translating into greater self‐reported quality of life (Kepka et al., 2013; Perales‐Montilla, García‐León, & Reyes‐del‐Paso, 2012; Petersen et al., 2008; Schou, Ekeberg, & Ruland, 2005). Therefore, studying the relationship between optimism and self‐related health in people with T2D may provide valuable information about the possible protective role of optimism in people living with a chronic disease.

The daily pattern of cortisol release is also important for health (Kondratova & Kondratov, 2012), and several parameters of circadian cortisol release have been employed, such as total cortisol output, cortisol slope across the day, and the cortisol awakening response (CAR). The CAR is a sharp rise in cortisol levels in the first 30 min after waking (Stalder et al., 2016), which has been related to multiple psychosocial factors: for example, the CAR has been positively related to job stress and general life stress, and negatively associated with fatigue, burnout, or exhaustion (Chida & Steptoe, 2009). However, the relationship between the CAR and positive psychological factors such as optimism is less consistent. Nevertheless, research by Jobin, Wrosch, and Scheier (2014) found that older people with low optimism had higher daily cortisol values and a lower CAR on the days that they reported higher stress. Similarly, studies with healthy older (Endrighi et al., 2011) and middle‐aged adults (Lai et al., 2005) reported that high optimism was related with lower CAR, but not with cortisol decline of the day or total cortisol output.

In the present study, we expanded on our previous investigation to assess the role of optimism on psychophysiological responses to stress in people with T2D. Taking into account that optimism is a protective trait, it is plausible that individuals with T2D who have high levels of optimism could have a better pattern of physiological stress responses; that is, their pattern of response more closely resembles the profile of stress responsivity in healthy individuals. So, we hypothesized that greater optimism would be related to heightened BP reactivity and better poststress recovery in people with T2D. No relationship was expected between optimism and acute cortisol response to stress, in line with previous research in older samples (Endrighi et al., 2011; Puig‐Perez et al., 2015). However, we did hypothesize that greater optimism would be inversely associated with cortisol output over the day. Finally, in line with previous studies, we expected that optimism would be positively related to better self‐related health (Kepka et al., 2013; Perales‐Montilla et al., 2012; Petersen et al., 2008; Schou et al., 2005). Our analyses took into account covariates that might potentially moderate psychophysiological responses in this population.

## Method

### Participants

The participants recruited for the present study were part of larger trial comparing stress responsivity in healthy individuals and people with T2D (Steptoe et al., 2014). All participants gave full informed consent to take part in the study, and the National Research Ethics Service granted ethical approval. A total of 140 participants with doctor‐diagnosed T2D were recruited between March 2011 and July 2012 from diabetes outpatient and primary care clinics in London. No respondents had a history of coronary heart disease, inflammatory diseases, allergies, or mood disorders. They were instructed to avoid the use of antihistamine or antiinflammatory medication in the 7 days prior to the study, and were instructed not to consume alcohol or practice intense exercise on the evening before the test, and to avoid caffeinated beverages and smoking during the 2 h prior to the testing session.

### Psychological Measures

We measured optimism using the 10‐item Life Orientation Test‐Revised (LOT‐R), a widely used measure of optimism trait that evaluates generalized positive or negative expectancies in life (Scheier, Carver, & Bridges, 1994). Participants were asked to indicate the extent of their agreement with each item (e.g. “I'm always optimistic about my future”) from 0 (*strongly disagree*) to 4 (*strongly agree*). Six items are used to derive the optimism score, so ratings can range from 0 to 24, with higher scores indicating greater optimism. The remaining four questions on the LOT‐R are filler items. The internal consistency (Cronbach's α) was .83 in this sample. This questionnaire was completed prior to the laboratory stress testing session. Subjective stress was assessed before and after stress (immediately poststress, 45 and 75 min after stress) using a 7‐point rating scale, with higher values indicating greater perceived stress.

### Other Measures

We included household income as an indicator of socioeconomic status, and the participants were categorized into low (< £20,000), medium (£20,000–£40,000), and high (> £40,000) income groups. Education was categorized into less than high school, high school or equivalent, and college or higher. The participants also reported whether they were in paid work at the time of stress testing. Ethnicity, smoking status, and medication were also recorded. Specifically, medication was divided into six categories: oral diabetic medication (metformin, etc.), insulin and other injected medication, aspirin, β blockers, other hypertensive medication (e.g., angiotensin‐converting enzyme inhibitors), and statins. Health status was measured using the 36‐item Short‐Form Health Survey (SF‐36; Ware & Sherbourne, 1992). Eight dimensions of functioning are assessed on the SF‐36, but for the purposes of this study the Physical Component Summary (PCS) and the Mental Component Study (MCS) were computed. Scores were coded and transformed to a scale where 0 = *worst possible health* and 100 = *best possible health*. The internal consistency (Cronbach's α) of the PCS was 0.91 and the MCS was 0.80 in this sample.

### Mental Stress Tasks

Two 5‐min behavioral tasks were used to induce mental stress in the laboratory. The tasks were administered in a random order. One task was the Stroop color‐word interference task, which consisted of successive presentations of target color words printed in an incongruous color. The other task was a mirror tracing task, in which the participant has to trace a star that could only be seen in mirror image using a mental stylus. If the respondent put the stylus outside of the star, the device emitted a loud beep (Lafayette Instruments Corp., Lafayette, IN) and a mistake was registered. Participants were told that the average person could complete five circuits of the star in the allocated 5‐min period. We selected these tasks because they have been shown previously to elicit similar appraisals of involvement and engagement from people across the social gradient and have been used in previous studies by our group (Steptoe et al., 2002).

### Procedure

Sessions were held in the morning or in the afternoon, in a light‐ and temperature‐controlled laboratory. At the beginning of the session, objective measures of height and weight were obtained, and body mass index (BMI) was computed. Systolic BP (SBP), diastolic BP (DBP), and HR were continuously monitored using a Finometer device (TNO‐TPD, Biomedical Instrumentation, Amsterdam, The Netherlands). After 30 min resting, the baseline cardiovascular values were measured in the last 5 min of the rest period, and respondents provided a baseline rating of subjective stress and a saliva sample for the assessment of cortisol. Participants then completed the two 5‐min mental stress tasks in a random order. SBP, DBP, and HR were measured with 5‐min recording periods during each of the tasks, and subjective stress and a second saliva sample were taken immediately after the tasks. Further cardiovascular measurements and subjective stress ratings were obtained at 45 and 75 min after the stress exposure. Additional saliva samples were collected at 20, 45, and 75 min after stress to assess cortisol responses.

Additionally, saliva samples were collected over a typical day in order to measure cortisol concentration over the day. Participants collected five saliva samples using Salivettes (Sarstedt) at waking, 30 min later, and then within three 30‐min time windows in the morning (10:00–10:30), afternoon (16:00–16:30), and evening (20:00–20:30). They were instructed not to eat, drink tea or coffee, or smoke in the 30 min before sample collection. Violations of this protocol and sample timing were recorded in a log. For the majority of people, cortisol sampling was carried out on the day after the laboratory visit. Saliva samples were stored at −20 degrees before analysis using a time‐resolved immunoassay with fluorescence detection at the University of Dresden. Samples were analyzed in duplicate; the intraassay and interassay coefficients of variation were less than 8%. Overall, 10 measures of cortisol were collected in the study, five from saliva samples taken in the laboratory (baseline, immediately, and 20, 45, and 75 min after stress), and five samples collected over a typical day (at waking, 30 min later, 10:00–10:30, 16:00–16:30, and 20:00–20:30).

### Statistical Analysis

SBP, DBP, and HR were averaged into 5‐min means at baseline, mean values of the two mental stress tasks, and at the two recovery periods (45 and 75 min after stress). Cortisol values were log‐10 transformed before analysis because of a skewed distribution. Subjective stress, SBP, DBP, and HR were analyzed across four trials (baseline, task, and 45 min and 75 min after stress). Laboratory cortisol was analyzed across five trials in the lab (baseline, task, and 20 min, 45 min, and 75 min after stress). Repeated measures analysis of variance (ANOVA) was used to test the responses to mental stress (subjective stress, SBP, DBP, HR, and laboratory cortisol), and main effects were followed up with post hoc tests using LSD (least significant difference). Repeated measures ANOVA was also used to analyze the profile of cortisol over the day in five samples (at awakening, 30 min after awakening, 10:00–10:30, 16:00–16:30, and 20:00–20:30).

We computed measures of reactivity (mental stress task minus baseline values) and recovery (45 min minus baseline values) for SBP, DBP, HR, and stress perception. The area under the curve with respect to the ground (AUCg) was calculated to test the cortisol output across the laboratory session (Pruessner, Kirschbaum, Meinlschmid, & Hellhammer, 2003). For cortisol over the day, we computed total output (AUCg) using the method described by Pruessner et al. (2003). Since the sample obtained 30 min after waking may distort the computation of total cortisol output, we measured the AUCg both using all samples (Day‐AUC_t_) and after excluding the sample taken 30 min after waking (Day‐AUC_ds_). The CAR was calculated by subtracting the awakening cortisol concentrations from the 30 min postawakening sample.

Associations with optimism were analyzed using multivariable linear regression on cardiovascular baseline levels, reactivity and recovery measures, stress perceptions, and the measures of cortisol output in the laboratory and over the day. Optimism was entered into the regression models along with age, sex, smoking status, and baseline values (laboratory cortisol, HR, SBP, and DBP) to test associations with physiological stress responses. Stress responses differ by sex in some studies (Kudielka, Buske‐Kirschbaum, Hellhammer, & Kirschbaum, 2004; Steptoe et al., 2002), and also sex differences in optimism have been reported (Helliwell, Layard, & Sachs, 2012). Sex was therefore included as a covariate in all analyses along with age. Smoking is known to impact physiological stress responses (Evans et al., 2012; Phillips, Der, Hunt, & Carroll, 2009), so smoking was controlled for in all analyses. Additionally, for laboratory cortisol, time of testing was entered as a covariate in case there were differences between the morning and afternoon. Optimism was entered into the regression models along with age, sex, smoking status, time of awakening, and day of sampling (week or weekend day) in the analyses of cortisol over the day. Delays between waking and taking the first saliva sample can distort the CAR (Dockray, Bhattacharyya, Molloy, & Steptoe, 2008); we excluded cases when the delay was more than 15 min after waking in the analysis of the CAR. However, we did not find a difference in the association between the CAR and optimism after excluding such individuals, so the complete study sample was used in the final analyses. Finally, partial correlations were performed to test the relationship between optimism and physical and mental health status (SF‐36 scales) taking account of age, sex, BMI, and household income. Covariates for these analyses were chosen after preliminary analyses in which we checked their relationship with physiological responses assessed in the study or/and health status as measured on the SF‐36, as well as research evidence of their relationship in previous studies (e.g., Cohen, 1996; Direk, Newson, Hofman, Kirschbaum, & Tiemeier, 2011; Ettner, 1996; Roy, Steptoe, & Kirschbaum, 1994; Stalder et al., 2016; Yan et al., 2004). Sensitivity analyses adding medication usage across multiple categories as an additional set of covariates did not change the pattern of results between optimism or the SF‐36 and physiological responses. Therefore, medications were not included as covariates in the final models presented in this manuscript.

One participant did not complete the LOT‐R, so was excluded from all the analyses. We used the Greenhouse‐Geisser procedure when the requirement of sphericity in the repeated measures ANOVA was violated. All *p* values reported are two‐tailed, the level of significance was set at *p* < .05 and 95% confidence interval (CI). We used SPSS 22.0 to perform the statistical analysis.

## Results

### Participant Characteristics

The sample consisted of 140 participants (88 men) with doctor‐diagnosed T2D. Participant characteristics are detailed in Table 1. It can be seen that the majority of respondents were white men, with an average age of 63.71 years old, and were typically overweight or obese (BMI average 30.75) with a modest household income (< £20,000). With regard to medication usage, 11% of the sample was using injectable antidiabetic drugs, and 11.8% were taking β blockers. Optimism scores averaged 14.43 in this sample, with scores ranging from 1 to 24. Optimism was related to age (*r* = .178, *p* = .036) and household income (*r* = .239, *p* = .006), but not with smoking status, BMI, or sex (all *p*s > .228).

**Table 1:** Participant Characteristics

Characteristics		
Age, *M* (*SD*)	63.71	7.004
Sex, *n* (%), % men	88	62.9
Ethnicity, *n* (%), % white	112	80.0
Current smoker, *n* (%), % smoker	20	14.3
Body mass index, *M* (*SD*), kg/m^2^	30.75	5.72
Household income, *n* (%)		
< £20,000	57	42.9
£20,000–£40,000	38	28.6
> £40,000	38	28.6
Education, *n* (%)		
Less than high school	37	26.8%
High school	14	10.0%
College or higher	87	63.0%
HbA1c, *M* (*SD*)	7.25	1.42
Paid work, *n* (%), % yes	62	44.3%
Injectable antidiabetic and insulin, *n* (%)	15	11.0
Oral antidiabetic, *n* (%)	109	80.1
β blockers, *n* (%)	16	11.8
Antihypertensive, *n* (%)	96	70.6
Cholesterol lowering, *n* (%)	106	77.9
Aspirin, *n* (%)	48	35.3
Other	62	45.6
Life Orientation Test‐R, *M* (*SD*)	14.43	4.32

*Note*. *M* = mean; *SD* = standard deviation; HbA1c = glycated hemoglobin.

### Psychophysiological Response to Stress

Repeated measures ANOVAs showed a main effect of time in SBP, DBP, and HR (all *p*s < .001). The exposure to stress provoked a significant increase in SBP, DBP, and HR from baseline, decreasing toward baseline in the poststress period (all *p*s < .001). Cortisol concentrations were highest at baseline, decreasing across the session until 45 min after the stress tasks (all *p*s < .001; see Table 2).

**Table 2:** Responses Across the Laboratory Session

	Baseline *M* (*SD*)	Stress task *M* (*SD*)	20 min *M* (*SD*)	45 min *M* (*SD*)	75 min *M* (*SD*)
SBP mmHg	126.08 (13.55)	149.35 (20.58)		134.24 (20.32)	137.05 (17.02)
DBP mmHg	71.74 (10.15)	84.25 (12.51)		78.04 (14.89)	79.51 (13.80)
HR beats/min	71.78 (12.36)	76.34 (12.23)		70.18 (12.23)	70.15 (11.94)
Stress perception	1.50 (0.90)	4.50 (1.52)		1.53 (0.93)	1.43 (0.93)
Cortisol nmol/mL	10.03 (5.34)	8.74 (4.35)	7.74 (3.92)	6.89 9(4.02)	7.17 (5.48)

*Note*. SBP = systolic blood pressure; DBP = diastolic blood pressure; HR = heart rate.

Stress perceptions peaked during tasks (*p* < .001), then returned to baseline in the poststress period.

### Cortisol Output Over the Day

As expected, there was a main effect of time in the analysis of cortisol sampled over the day (*p* < .001). Cortisol concentration averaged 19.98 ± 11.98 on waking, increasing to 26.60 ± 14.81 30 min later (*p* < .001). After that, the cortisol concentration decreased over the course of the day to its lowest level in the evening (mean 5.48 ± 5.9, *p* < .001).

### Optimism and Responses to Mental Stress

Regression analyses showed no relationship between optimism and baseline SBP (*p* > .805) or DBP (*p* > .757) with and without controlling for covariates. Regression analyses showed that optimism was associated with higher SBP (β = 0.185, CI = 0.041–1.325, *p* = .037) and DBP (β = 0.176, CI = 0.003–0.568, *p* = .047) responses to stress (Table 3). Thus, more‐optimistic people with diabetes were more reactive in terms of BP after controlling for age, sex, smoking status, and baseline levels. There was no association between optimism and SBP and DBP recovery, HR reactivity or recovery, or with laboratory cortisol AUCg (all *p*s > .175).

**Table 3:** Summary of Regression Analyses with Optimism and Physiological Stress Response

	SBP				DBP				HR				Cortisol	
	Reactivity *R* ^2^ = .041 *p* = .356		Recovery *R* ^2^ = .062 *p* = .135		Reactivity *R* ^2^ = .042 *p* = .345		Recovery *R* ^2^ = .024 *p* = .674		Reactivity *R* ^2^ = .074 *p* = .071		Recovery *R* ^2^ = .060 *p* = .145		AUCg *R* ^2^ = .580 *p* < .001	
	β	*p*	β	*p*	β	*p*	β	*p*	β	*p*	β	*p*	β	*p*
Age	0.050	.575	−0.006	.945	0.057	.534	−0.037	.687	−0.056	.527	0.004	.968	−0.001	.986
Sex	−0.029	.744	0.151	.087	0.014	.881	0.047	.608	−0.089	.301	0.091	.293	−0.079	.204
Smoking	0.015	.866	0.088	.321	−0.051	.559	0.042	.638	−0.116	.175	−0.147	.089	0.090	.156
Baseline	−0.016	.857	−0.158	.077	0.036	.701	0.074	.437	−0.216	.014	−0.151	.086	0.188	.002
Time of testing	–	–	–	–	–	–	–	–	–	–	–	–	0.694	<.001
Optimism	**0.185**	**.037**	0.118	.175	**0.176**	**.047**	0.109	.220	0.115	.180	0.038	.664	0.075	.225

*Note*. Bold text denotes statistically significant results. SBP = systolic blood pressure; DBP = diastolic blood pressure; HR = heart rate.

Regression analyses showed no relationship between optimism and increases in perceived stress in response to tasks (β = −0.083, CI = −0.090–0.027, *p* = .287) or with the decrease in stress ratings 45 min poststress (β = −0.021, CI = −0.042–0.032, *p* = .781).

### Optimism and Cortisol Over the Day

There was a significant negative association between optimism and cortisol output over the day, with (Day‐AUC_t_: β = −0.261, CI = −7.339 to −1.387, *p* = .004) and without including the 30 min after waking sample in the model (Day‐AUC_ds_: β = −0.283, CI = −6.072 to −1.521, *p* = .001) after controlling for age, sex, educational level, BMI, smoking status, time of awakening, and day of sampling (Table 4). Regression analyses showed no significant relationship between optimism and the CAR after controlling for covariates (*p* = .531).

**Table 4:** Summary of Regression Analyses with Optimism and Daily Cortisol

	Day‐AUC_t_ *R* ^2^ = .144 *p* = .014		Day‐AUC_ds_ *R* ^2^ = .194 *p* = .001		CAR *R* ^2^ = .047 *p* = .560	
	β	*p*	β	*p*	β	*p*
Age	0.015	.878	0.023	.803	0.031	.755
Sex	−0.102	.266	−0.100	.254	0.046	.623
Smoking	−0.029	.748	−0.056	.524	0.039	.675
BMI	0.123	.206	0.192	.039	−0.003	.976
Day of testing	−0.171	.059	−0.182	.036	−0.093	.310
Time of awakening	−0.119	.189	−0.133	.126	−0.163	.078
Optimism	**−0.261**	**.004**	**−0.283**	**.001**	0.058	.531

*Note*. Bold text denotes statistically significant results.

### Optimism and Self‐Reported Health Status

Partial correlations with age, sex, BMI, and household income as covariates showed significant relationships between optimism and SF‐36 total scores of physical and mental health. Optimism was positively related to better outcomes in physical (PCS) (*r* = .303, *p* < .001) and mental health (MCS) total scores (*r* = .355, *p* < .001).

## Discussion

This study investigated the relationship between optimism and cardiovascular, neuroendocrine, and psychological response to acute stress and cortisol secretion over the day in middle‐aged and older people with T2D. The behavioral tasks elicited marked increases in cardiovascular activity along with subjective distress, indicating that they were effective in stimulating acute stress responses. Our main finding was that people with T2D with low optimism showed blunted SBP and DBP responses to stress. No associations between optimism and cortisol response to acute stress and CAR were found, but the low optimism group showed higher diurnal cortisol concentrations and poorer health status, as measured with the SF‐36.

Agreeing with previous studies (Endrighi et al., 2011; Puig‐Perez et al., 2015), optimism was not related with the cortisol response to stress, but was related to cardiovascular responses to stress (SBP and DBP) in people with T2D. In contrast with Solberg Nes et al. (2005), we observed a relationship with SBP and DBP, but not with HR. It should be taken into account that participants of the present study had diagnosed T2D, and these individuals have previously been shown to have blunted SBP and DBP responses to stress when compared with healthy matched controls (Steptoe et al., 2014). Therefore, the results of the present study suggest that optimism in people with T2D is associated with heightened BP responses to stress, which is in line with stress responsivity observed in healthy individuals. Considering that people with T2D show blunted cardiovascular response to stress (Steptoe et al., 2014), our results contribute to the evidence for the protective effect of optimism in T2D, supporting the protective role of an optimistic perspective (Carver & Scheier, 1990; Carver et al., 2010; Scheier & Carver, 1993). That is, our results support the assumption that optimism helps people with T2D to preserve a better physiological adjustment to stress. It is plausible that this could make optimistic people with T2D less likely to develop common comorbid diseases. The stress response is an adaptive response to the challenges of the environment through the adjustment of multiple physiological systems, but repeated or sustained stimulation of these systems can disrupt dynamic responses to acute challenges or stress resulting in impaired stress reactivity and recovery (McEwen, 1998). For this reason, factors such as optimism that facilitate similar responsivity to stressful circumstances in people with T2D as in healthy individuals could help to reduce the consequences of impaired cardiovascular function, which is of importance as cardiovascular disease (CVD) is one of the most common comorbid problems in T2D (Grundy et al., 1999).

In keeping with our hypotheses and previous literature (Jobin et al., 2014), low optimism was related to higher diurnal cortisol pattern in people with T2D. Considering that T2D is characterized by heightened daily cortisol (Steptoe et al., 2014), the lack of optimism might accentuate disruption of daily cortisol release in this population. However, contrary to our hypothesis and several studies (Endrighi et al., 2011; Jobin et al., 2014; Lai et al., 2005), we did not find any relationship between the CAR and optimism. But, it should be taken into account that the above‐mentioned studies (Endrighi et al., 2011; Jobin et al., 2014; Lai et al., 2005) were conducted with healthy participants and not people with T2D, thus different results could be expected between optimism and daily cortisol in a diseased versus healthy population.

Finally, and agreeing with previous studies (Kepka et al., 2013; Perales‐Montilla et al., 2012; Petersen et al., 2008; Schou et al., 2005), optimism was related to better physical and mental subjective well‐being. Therefore, our results support that an optimistic point of view in T2D is associated with better ratings of subjective physical and mental health, as well as a pattern of stress responsivity closer to that of healthy individuals. Taking into account the damaging impact of chronic diseases on health‐related quality of life (Rothrock et al., 2010) and psychological well‐being (Wilsher, 2012), our results provide valuable information about the possible protective role of optimism in a diseased population.

The present study should be interpreted in the light of various limitations. Firstly, the sample recruited consisted of people with T2D without history of coronary heart disease from the London area, and the majority of participants were of white European origin. Thus, it is possible that these results may not apply to other groups. Furthermore, cortisol was only assessed over 1 day, which might have limited our ability to assess physiological functioning. This is a cross‐sectional study, and further longitudinal research is needed to understand how optimism is associated with cardiovascular and cortisol functioning in people with T2D over time. Finally, it is important to note that some studies investigate optimism and pessimism poles as distinct constructs (Herzberg, Glaesmer, & Hoyer, 2006; Lai et al., 2005; Puig‐Perez et al., 2015), whereas in the present study we assessed optimism as a continuous measure. As the optimism trait is based on historical experiences, it is possible that a third unmeasured variable accounted for the relationship. We did not have information on diabetes duration or age of diabetes onset, and the protective effect of optimism could differ depending on disease duration and severity. Optimism was not correlated with HbA1c or medication usage in this study (data not shown), which makes it unlikely that diabetes severity impacted the relationships presented in this paper. Nevertheless, this possibility cannot be completely excluded based on the information available.

Despite these considerations, the results suggest that SBP and DBP responses to stress are blunted in people with T2D with low optimism. Moreover, these people had higher cortisol concentrations over the day. Both blunted cardiovascular response to stress and heightened daily cortisol concentrations could result in future health problems such as CVD. However, further studies are required to confirm these pathways.
